# Real-time fMRI brain-computer interface: development of a “motivational feedback” subsystem for the regulation of visual cue reactivity

**DOI:** 10.3389/fnbeh.2014.00392

**Published:** 2014-11-25

**Authors:** Moses O. Sokunbi, David E. J. Linden, Isabelle Habes, Stephen Johnston, Niklas Ihssen

**Affiliations:** ^1^MRC Centre for Neuropsychiatric Genetics and Genomics, Institute of Psychological Medicine and Clinical Neurosciences, Cardiff School of Medicine, Cardiff UniversityCardiff, UK; ^2^Cardiff University Brain Research Imaging Centre (CUBRIC), Cardiff UniversityCardiff, UK; ^3^Department of Psychology, Swansea UniversitySwansea, UK

**Keywords:** brain-computer interface (BCI), hunger, visual cue reactivity, food craving, functional magnetic resonance imaging (fMRI), neurofeedback, self-regulation

## Abstract

Here we present a novel neurofeedback subsystem for the presentation of motivationally relevant visual feedback during the self-regulation of functional brain activation. Our “motivational neurofeedback” approach uses functional magnetic resonance imaging (fMRI) signals elicited by visual cues (pictures) and related to motivational processes such as craving or hunger. The visual feedback subsystem provides simultaneous feedback through these images as their size corresponds to the magnitude of fMRI signal change from a target brain area. During self-regulation of cue-evoked brain responses, decreases and increases in picture size thus provide real motivational consequences in terms of cue approach vs. cue avoidance, which increases face validity of the approach in applied settings. Further, the outlined approach comprises of neurofeedback (regulation) and “mirror” runs that allow to control for non-specific and task-unrelated effects, such as habituation or neural adaptation. The approach was implemented in the Python programming language. Pilot data from 10 volunteers showed that participants were able to successfully down-regulate individually defined target areas, demonstrating feasibility of the approach. The newly developed visual feedback subsystem can be integrated into protocols for imaging-based brain-computer interfaces (BCI) and may facilitate neurofeedback research and applications into healthy and dysfunctional motivational processes, such as food craving or addiction.

## Introduction

Recent advancements in real-time functional magnetic resonance imaging (fMRI) have been based on the increased availability of high-field (in excess of 1.5 tesla) MRI scanners, fast data acquisition sequences, improved real-time pre-processing, improved statistical analysis techniques and methods of data visualization and feedback. These developments have made the implementation of fMRI brain-computer interfaces (fMRI-BCI) and neurofeedback possible (Weiskopf, 2012). In fMRI-BCI, the self-regulation of brain activity in a target region can be achieved by providing real-time feedback of brain activation levels in that target region. While paradigms more commonly utilize feedback signals based on overall activity levels in a given region, more complex metrics of brain activation are being used based on multivariate statistical techniques (LaConte et al., 2007; Sitaram et al., 2008) or correlation of activity in several brain areas (Zilverstand et al., 2014).

### Neurofeedback and visual cue reactivity

In recent years an emerging but technically challenging field in neurofeedback research has been the pairing of feedback signals (and regulation instructions) with simultaneous visual stimulus presentation. Such approaches are especially interesting for research into neural and behavioral responses that are passively triggered by salient emotional or motivational cues, such as fear responses evoked by emotional pictures or craving elicited by drug-related pictures. The degree of drug cue-elicited neural activation within circuits of the orbitofrontal cortex (Ernst et al., 2014), the nucleus accumbens and medial prefrontal cortex (PFC; Wiers et al., 2014) has been shown to determine the degree of cue approach vs. avoidance behavior in alcohol dependent patients. Moreover, alcohol cues automatically evoke cognitive processes that are biased towards approaching the cue and correlate with drinking behavior in adolescents (Peeters et al., 2012). Neurofeedback training to control these emotional/motivational responses should thus aim at the down-regulation of blood oxygen level dependent (BOLD) responses, especially when the targeted neurocognitive process is considered to be dysfunctional. To date, however, most fMRI neurofeedback studies have trained participants to up-regulate activation of a target area. Further, a direct comparison between up- and down-regulation training of the same brain region (anterior insula) showed that down-regulation was more difficult to learn (Veit et al., 2012). In light of its promising clinical applications, this underscores the need for methodological development and research into the area of neurofeedback related to visual cue reactivity. Here we present a novel neurofeedback subsystem for the presentation of motivationally relevant feedback during exposure to appetitive pictures.

### The choice of feedback type

When setting up visual cue reactivity neurofeedback paradigms, researchers are confronted with several challenges. One relates to the choice of feedback type: With the simultaneous visual presentation of feedback information and task stimulus, there is a potential risk that monitoring the feedback stimulus distracts attention from the emotional/motivational cue which will confound any effortful down-regulation of the targeted neural response. Since the pioneering neurofeedback studies at the beginning of the last decade, various techniques have been employed to present visual feedback for the self-regulation of BOLD activation over time. Weiskopf et al. (2003, 2004) and deCharms et al. (2004) have used scrolling time series graphs and curves of BOLD activation in the region of interest (ROI) to provide immediate information to the participant. Sitaram et al. (2005) introduced the “thermometer” as a type of visual feedback for the display of brain activity. Positive BOLD activity, compared to a baseline period is shown as an increasing number of red “hot” bars, whereas negative BOLD activity is similarly shown in blue. The authors also introduced a virtual reality feedback system (Sitaram et al., 2005) where volunteers controlled a 3D animated character (a fish in water). Other visual feedback approaches include the presentation of functional maps of the brain (Yoo and Jolesz, 2002) or video-based feedback that has been used to train stroke patients to self-regulate ventromedial premotor cortex (Sitaram, 2007a).

Most studies using simultaneous visual stimulation during feedback-guided regulation have adopted the thermometer approach as described above but temporally separated the feedback presentation from the regulation/visual stimulation period (Posse et al., 2003; Li et al., 2013). In contrast to such delayed feedback, continuous/on-line feedback during regulation has the advantage that participants can adaptively test different mental strategies to optimize regulation. Only few studies have paired the presentation of pictorial cues with BOLD signal feedback in a simultaneous manner. Brühl et al. (2014) trained participants to down-regulate activity of the right amygdala while being exposed to negative emotional faces. During the 20-sec down-regulation blocks, a feedback stimulus reflecting activation levels of the amygdala was presented to the participants, showing two colored rectangles at the picture edges which color-coded activity levels in a similar way as the thermometer approach described above. Rather than using a peripheral feedback stimulus, Veit et al. (2012) embedded the thermometer inside the simultaneously presented emotional cues that consisted of blocks of aversive pictures. Also, Mathiak et al. (2010) provided a positive feedback through facial expression (smiling) when activity in the anterior cingulate cortex (ACC) increased and gradually returned to a neutral expression when the activity dropped.

Critically, such spatial or feature-based distinction between the feedback and the task stimulus can induce distraction or interference effects as seen in dual tasks. As a consequence, responses to the task stimulus (e.g., emotional picture) may be reduced, confounding the effects of effortful down-regulation. Here, we delineate a novel approach in which feedback-guided self-regulation is based on visual changes in the stimulus responsible for the targeted brain responses itself. Specifically, the present paradigm uses appetitive food pictures to evoke responses in brain circuits related to hunger and food craving. The success of regulating those responses during picture exposure in the neurofeedback session is represented as gradual changes in image size. Compared to the feedback stimuli used by Veit et al. and Brühl et al. our paradigm has the advantage that distracting/dual-task effects associated with monitoring the feedback stimulus during cue exposure are minimized. At the same time, by linking decreases in image size to successful down-regulation and image size increases to failure, respectively, the paradigm provides real motivational consequences of the participant’s regulation effort, i.e., the stimuli mimic avoidance behavior during successful down-regulation and approach behavior during unsuccessful down-regulation. Such behavioral/motivational relevance of the task can increase face validity of the neurofeedback training and may thus facilitate its therapeutic use in pathologies of motivational systems, such as obesity or addiction.

### The issue of habituation

Another challenge of visual cue reactivity neurofeedback is that repeated exposure to the same or similar visual stimulus will lead to a decrease of neural responses. Such effects have been well documented in brain imaging tasks using fMRI adaption or “repetition suppression” techniques, in which the reduction of BOLD responses associated with the repeated presentation of identical stimuli is used as a tool to characterize the neural representation of visual objects (Grill-Spector et al., 2006). Similar effects of neural adaptation can also occur in brain circuits involved in emotional processes. For instance, multiple presentations of threatening pictures will lead to a gradually weaker activation of the amygdala (Wright et al., 2001). In the present context of down-regulation of cue-induced BOLD activation patterns, it is thus crucial to control for such effects, as a gradual habituation effect over successive regulation blocks may be misinterpreted as reflecting successful regulation. In previous research such control measures have been implemented, for instance, by including a passive viewing condition (either within- or between-runs) where the same set of pictures and the feedback stimulus shown during regulation is repeated but participants are instructed not to regulate their brain responses (Brühl et al., 2014). Here, we took a similar approach by including “mirror” runs in the paradigm, in which participants were exposed to the same size sequence that was “produced” during a previous regulation block but instructed not to regulate target area activation.

### Computational translation and response range adaptation of the BOLD signal

All BCIs include a series of steps for converting the measured brain signal, such as percentage signal change, into commands (Linden, 2014). First, the relevant feature is extracted from the wealth of information that the measurement device picks up, ideally in real time. Secondly, the extracted feature needs to be converted into an output signal for the participant to use through a translation algorithm. Finally, the translation from the extracted feature of the brain signal to the output needs to be adaptive. Peoples’ individual neurophysiological responses vary widely yet all must ultimately appear in an identical summary form, for their use, projected onto a computer screen. Thus the conversion has to be adapted to an individual’s response range. This adaptation to the original signal also has to take into account the fluctuation of the signal over time and the improvement with training as well as an individual’s training capacity. For example the duration of the training has to be adjusted to psychological factors like motivation and fatigue, which again corresponds to well-known general principles from teaching and training.

A well designed feedback system is an important criterion in successfully training participants to self-regulate their BOLD response. Contingent feedback following the participant’s response with minimum lag and with reliable information content pertaining to task success improves learning (Sitaram et al., 2008). As detailed below, in the current project we developed a solution for steps two (translation algorithm) and three (adaptation) of the motivational BCI approach outlined above.

## Materials and methods

### fMRI brain-computer neurofeedback architecture

**Figure 1:**
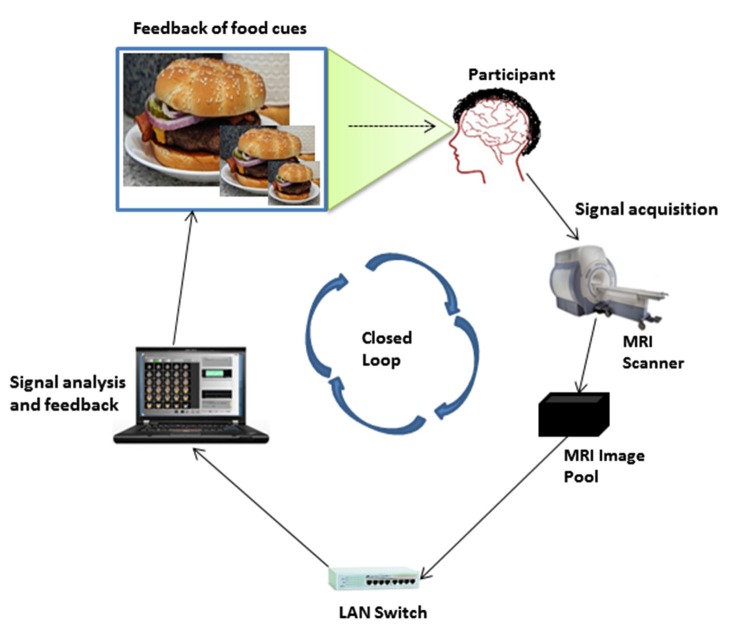
**fMRI BCI architecture**.

Figure 1 describes the fMRI-BCI architecture for neurofeedback training at our center. Our architecture is a closed-loop system with the following major subsystems; signal acquisition, signal analysis and signal feedback. In Figure 1, the MRI image pool is the end point of the signal acquisition subsystem and is connected to the signal analysis and signal feedback subsystems through a local area network (LAN). The signal analysis subsystem communicates with the signal feedback subsystem and they both reside on the same computer. The signal feedback subsystem is connected to the projector screen, where the feedback signal is presented.

At the signal acquisition subsystem, localized brain activity of the participant while viewing images on the projector screen is measured by fMRI using a BOLD sequence; a contrast is then made between the signal elicited by the target stimuli (food pictures) and neutral control stimuli (household objects in localiser run, see below), or a fixation baseline (regulation runs). A 3 T whole body scanner (General Electric, Milwaukee, USA) with an 8-channel head coil is used. The parameterisation of the BOLD sensitive echo planar imaging (EPI) sequence is as follows: TR = 2 s, TE = 45 ms, flip angle = 80°, 30 slices, FOV = 192 mm, image matrix 64 × 64, in-plane voxel size = 3 mm × 3 mm, slice thickness = 4 mm and a gap of 1 mm. Image reconstruction, distortion correction and image averaging are performed on the MRI scanner computer and stored in the MRI image pool.

The signal analysis subsystem is performed using Turbo-Brainvoyager (TBV) version 3.0 (Brain Innovation, Maastricht, The Netherlands; (Goebel, 2001)). TBV retrieves reconstructed fMRI images from the MRI pool via the LAN and performs online 3D motion correction, temporal filtering, spatial smoothing, spatial normalization and online statistical analysis calculating beta parameters from an incremental general linear model (GLM) based on the predictors of interest (e.g., food vs. neutral pictures). A static ROI is selected by drawing an area on the functional map (3D BOLD signal) computed in TBV. All supra-threshold voxels (according to an investigator-chosen statistical threshold between 2 and 3) are included in the target area for signal extraction. Average BOLD signal values (betas) from the target area are extracted by TBV and stored in a continuously updated real-time protocol file (rtp file).

The signal feedback subsystem retrieves the rtp files, processes and analyses the BOLD signal values contained within. Feedback is presented to the participant in real-time as food image sizes corresponding to the percentage change in BOLD signal values of the ROI during food picture presentation relative to fixation baseline using a moving average across three consecutive TRs. Feedback is presented to the participant with a delay that depends on the time for signal acquisition, signal analysis and signal feedback processing. Minimizing the delay is critical for volitional control (Sitaram et al., 2007b). Applying the general experience from operant conditioning experiments, the maximum delay for successful learning of biological responses is 2 min (Yoo et al., 2004). The design and implementation of the visual feedback subsystem for the regulation of hunger or food craving is described in the following sub-sections.

### Design

The visual feedback subsystem involves two types of functional imaging run; the neurofeedback and mirror/control runs. The present paradigm involves four runs of each type presented in an alternating order. The neurofeedback runs are the experimental runs, where the participants learn to control their fMRI signal in a block design with periods of rest followed by periods of down-regulation. During the rest blocks, a fixation cross is displayed for 20 s. During the down-regulation block, also 20 s in duration, one food image is presented which varies in size dependent on the percent fMRI signal change, during the block, relative to the preceding fixation block. There are five rest blocks and four down-regulation blocks in the entire display sequence of the neurofeedback run totalling a run length of 180 s. During the down-regulation block, the size of the food image is updated every TR (2 s) leading to a consecutive display of 11 different image sizes in total. Image sizes can vary between 10% and 100% of the original image size (1013 by 760 pixels) that are distributed across the whole size range. The size of the food image displayed at the first TR of each down-regulation block is set to 50% of the maximum image size, which corresponds to the percent signal change (PSC) at the first TR (FPSC) relative to the preceding rest block. Here, the FPSC is taken as the reference scaling point for the calibration of subsequent PSCs. The increase or decrease in image size calibration of the PSC is described using the following calibration algorithm:

def PSCDisplay(PSC):

if PSC < FPSC - 100:

Display 10% of the image size

elif FPSC - 100 <= PSC < FPSC - 75:

Display 15% of the image size

elif FPSC - 75 <= PSC < FPSC - 50:

Display 20% of the image size

elif FPSC - 50 <= PSC < FPSC - 25:

Display 30% of the image size

elif FPSC - 25 <= PSC < FPSC:

Display 40% of the image size

elif PSC == FPSC:

Display 50% of the image size

elif FPSC + 25 >= PSC > FPSC:

Display 60% of the image size

elif FPSC + 50 >= PSC > FPSC + 25:

Display 70% of the image size

elif FPSC + 75 >= PSC > FPSC + 50:

Display 80% of the image size

elif FPSC + 100 >= PSC > FPSC + 75:

Display 90% of the image size

elif PSC > FPSC + 100:

Display 100% of the image size

retun Display

This manner of calibrating the PSCs ensures that the effect of the FPSC is reflected in all the image sizes and adapts the feedback subsystem to peoples’ individual neurophysiological response range. Each neurofeedback block uses as its feedback signal a different food image from a pool of 16 pictures selected at random. By default the range of the response is set to a 1% deviation from baseline (upwards or downwards), which implies that a 1% up-regulation will result in presentation of the full-size image and 1% down-regulation in presentation of the smallest image. Further up- or down-regulation will then not be reflected in further in- or decreases of the image size. However, the gain of the conversion from the extracted brain signal feature (% BOLD signal change) to image size can be set freely to reflect an individual’s response range, and can be adjusted adaptively to reflect dynamic changes in self-regulation ability. The food images were taken from the International Affective Picture System (Lang et al., 2005) and the Internet.

**Figure 2:**
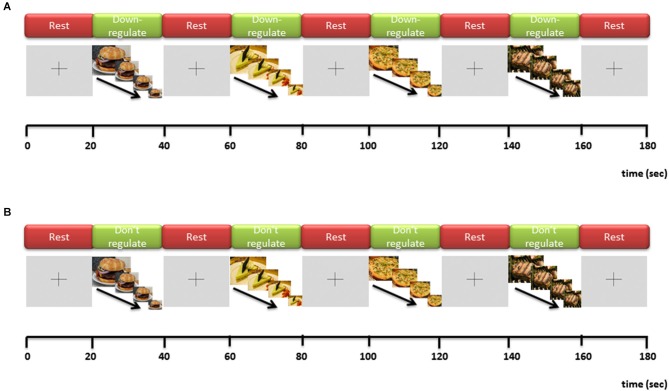
**Temporal structure of one neurofeedback run (A) and one mirror run (B)**.

After each neurofeedback run, an associated mirror/control run is presented to the participant. In the mirror run the exact same picture (size) sequence (using the same picture exemplar) is repeated that was shown in the preceding neurofeedback run. The only difference is that during the mirror run participants are instructed NOT to regulate their brain responses during exposure to the food images but should passively watch the image (size) sequences instead. By this means, the mirror run can serve as a perceptual control: when BOLD responses from the mirror run are (offline) subtracted from BOLD responses in the neurofeedback run, any decrease in brain activation (in the target ROI or elsewhere in the brain) *cannot* be attributed to differences in physical stimulation—which may include diminishing brain activation to motivational cues as a result of habituation over time/successive runs—but will likely reflect the down-regulation effort. Importantly, each mirror run is always presented *after* its corresponding regulation run showing the same picture exemplar and size sequence. Any habituation-related reduction of brain responses caused by repeating the same stimulation in the mirror run thus *cannot* lead to an erroneous detection of down-regulation success (difference regulation—mirror) as it works in the opposite direction. Figures 2A,B depicts the temporal structure of the neurofeedback and mirror runs.

### Implementation

**Figure 3:**
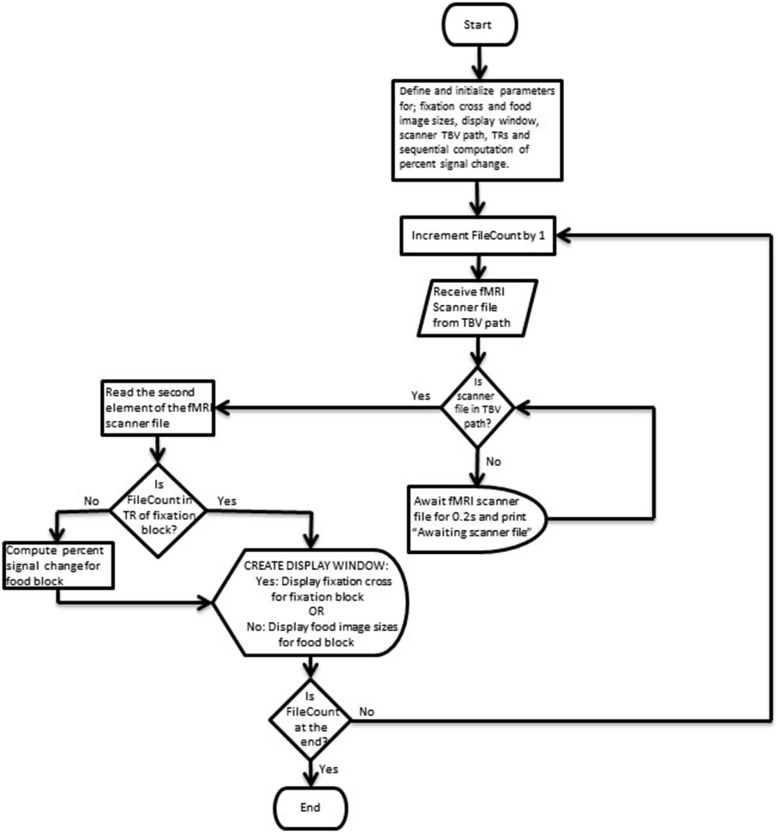
**Flow-chart depicting the execution path of the neurofeedback run**.

**Figure 4:**
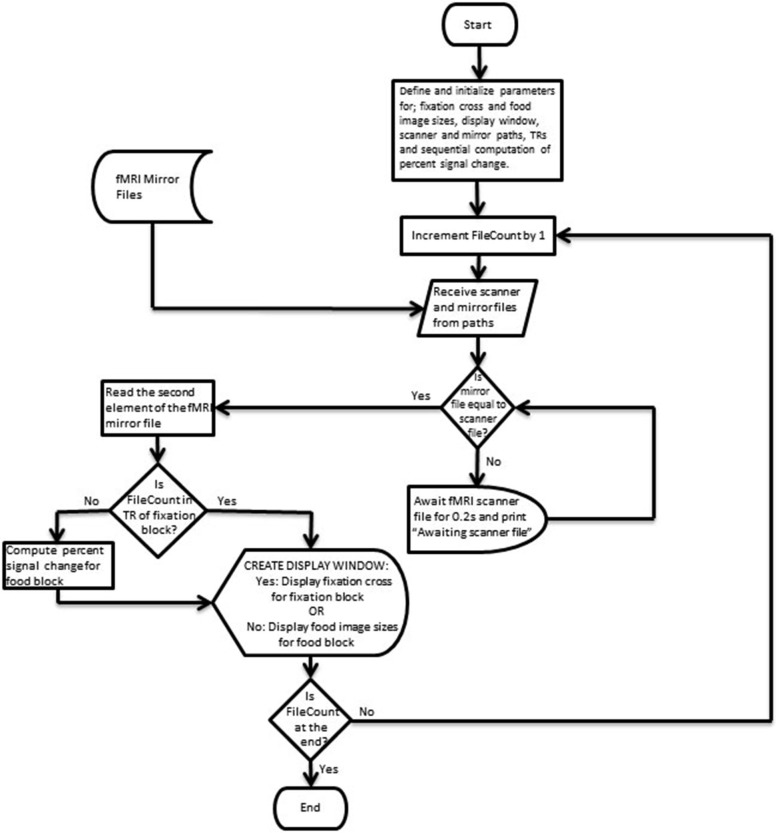
**Flow-chart depicting the execution path of the mirror run**.

Figures 3, 4 depict flow-charts of the execution path of the neurofeedback and mirror runs respectively. The flow-charts were implemented by writing the sequence of executed events in the Python programming language and executed using the PsychoPy graphical user interface (Peirce, 2007). During the neurofeedback run, the signal feedback subsystem accesses the average BOLD signal values of the ROI time courses (in the rtp files) computed by TBV and presents the feedback display in Figure 2A. The mirror run presents a visually identical feedback display (see Figure 2B) which uses the average BOLD signal values of the neurofeedback run stored in a separate directory (termed “mirror files”). The mirror files are processed and displayed in synchronization with the average BOLD signal values of the ROI time courses (rtp files) computed by TBV during the mirror run (scanner files).

### Pilot study testing the feasibility of “motivational neurofeedback”

Data from 10 female participants (mean age *M* = 21.40 years, Standard Deviation (SD) = 2.27) were acquired to test whether the newly developed motivational neurofeedback paradigm can be successfully used to down-regulate brain activation in response to appetitive food pictures. Participants were asked not to eat for 4 h before the scanning session to increase motivational brain responses related to hunger or food craving. Written informed consent was obtained in accordance with the local ethics committee prior to the start of the study.

The experiment began with the functional localizer run followed by an alternating sequence of four neurofeedback and four mirror runs. During the localizer run the participants passively viewed five blocks of food pictures and five blocks of pictures showing neutral household objects in alternating order. The picture blocks contained five pictures randomly selected from the relevant category, presented for 2 s each. Blocks were interleaved with a 10 s fixation period (with the exception of an initial fixation period at the start of the experiment which was 12 s in duration), resulting in a total run length of 222 s. The sequence of four neurofeedback and mirror runs were presented using the trial/run structure described above. Due to technical problems, for three participants only three neurofeedback/mirror runs could be acquired. For ROI analyses testing the feasibility of down-regulation of the target area in the neurofeedback runs, we used the pre-processed functional images created by the TBV software, which were co-registered to individual high-resolution T1-weighted anatomical images and spatially normalized to Talairach space. Individual volume time courses were spatially smoothed using a kernel with a full width at half maximum (FWHM) of 4 mm and temporally filtered with a high pass filter of 2 cycles/time course. For each run and participant separately, mean beta estimates in the target area were then extracted based on individual whole-brain GLMs and three different predictors for BOLD signal changes during (i) mirror, (ii) regulation (neurofeedback); and (iii) rest blocks. Table 1 shows the associated brain regions, mean Talairach coordinates and size of activation clusters selected as target areas for neurofeedback-guided down-regulation in the pilot sample.

**Table 1:** **Associated brain region (Left/Right), mean Talairach coordinates and size (1 × 1 × 1 mm^3^ voxels) of activation clusters selected as target areas for neurofeedback-guided down-regulation in the pilot sample**.

Participant	Region	Mean coordinates *(x, y, z)*	Size (voxels)	Mean beta difference regulation-mirror
01	Amygdala (L & R)	−2, −4, −14	1853	−0.18
02	Amygdala (R)	16, 2, −16	3185	−0.51
03	Amygdala (R)	28, −4, −12	3488	−0.25
04	Amygdala (L)	−27, −6, −20	1530	0.06
05	Amygdala (L & R)	4, −5, −14	3887	−0.10
06	Putamen (R)	−22, 14, -5	1534	−0.28
07	Insula (L)	−32, −1, 6	3979	−0.16
08	Caudate (R)	11, 0, 17	4637	−0.11
09	Thalamus (R)	24, −24, 3	1128	−0.14
10	Parahippocampal Gyrus (L)	−19, −30, −4	878	0.02

*Target areas were functionally selected using a localizer scan with food and neutral pictures. The table also includes mean beta differences for the regulation/neurofeedback vs. mirror/passive viewing condition across runs. Negative values indicate successful down-regulation of target area activation during neurofeedback runs*.

## Results and discussion

Based on a whole brain GLM analysis for the contrast between food and neutral pictures during the localiser run we visually selected for each participant individually a target area (ROI) encompassing the cluster showing the strongest response in the statistical activation maps (see Table 1). The feedback signals (and its corresponding picture sizes) in the subsequent neurofeedback runs were computed as the average percent signal change from all significantly activated voxels within a rectangle drawn over the target region across three axial slices. We constrained the selection to regions with a known involvement in emotional-motivational processes and to non-visual brain areas.

**Figure 5:**
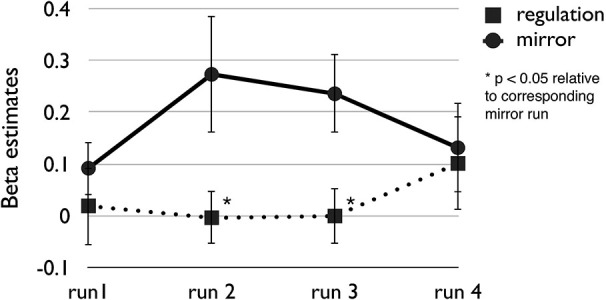
**Mean beta weights reflecting brain activation of individually selected target areas in 10 pilot participants during four consecutive neurofeedback (regulation) and mirror (passive viewing) runs**. Error bars indicate standard errors of the mean.

During the neurofeedback run participants were asked to reduce the size of the food images displayed on the projector screen by reducing the average fMRI signal strength in the target ROI. During the mirror run the participants were instructed to passively view the food image sequence repeated from the previous neurofeedback run. Figure 5 shows mean beta estimates for the pilot sample, separated for the four consecutive regulation and mirror runs. Paired *t*-tests showed that the regulation condition led to significantly lower target area activation in run 2, *t*_(9)_ = 2.49, *p* = 0.034, and in run 3, *t*_(9)_ = 2.77, *p* = 0.022. Activation levels were not significantly different between regulation and passive viewing in run 1 and run 4, *t*s < 1.0. However, as indicated in Figure 5 this was caused by increased activation in the mirror condition in the first and fourth run while target area activation during regulation was low throughout the session, with beta estimates not exceeding 0.1. Across runs, 8 out of 10 participants successfully reduced target area activation during neurofeedback, showing lower mean beta values for the regulation vs. passive viewing condition (see Table 1). To summarize, pilot data suggests that our paradigm enables participants to successfully down-regulate brain areas involved in processing motivational cues, such as appetitive food pictures. These results may be relevant to the increasing interest in the combination of Pavlovian and instrumental techniques in neurofeedback research (Mendelsohn et al., 2014).

### Motivational neurofeedback—relevance for clinical applications

Providing motivationally relevant neurofeedback through changes in stimulus size may also provide an avenue to help patients gain control over dysfunctional motivational processes, such as craving elicited by environmental cues in substance dependence. It is well known that visual cue reactivity, and specifically craving responses to drug-related cues (e.g., in the media or during social interactions) are a major determinant of relapse after treatment of addiction (Weiss, 2005). Moreover, maladaptive brain responses to visual drug cues can be identified in early stages of addictive disorders (Ihssen et al., 2011). Identifying a suitable neurofeedback approach that can help to alter these activation patterns directly would thus provide an important clinical tool complementing traditional psychological and pharmacological interventions. A few studies have begun to test the effects of neurofeedback in the context of addiction. For instance, Li et al. (2013) and Hanlon et al. (2013) trained treatment-seeking smokers to regulate brain responses in craving-related brain areas ACC and PFC during exposure to smoking-related pictures. Pictures were presented in blocks of 22 s during which participants were asked to regulate ACC/PFC activity, followed by a thermometer feedback shown for 4 s. However, these studies did not control for habituation effects as described above, presenting regulation runs always after passive viewing control runs. Moreover, feedback was implemented with a delay. The present approach overcomes these limitations. It may also be especially suitable in an applied setting as it has high face validity and presents to the participant visible, quasi-behavioral consequences of his/her mental regulation effort: Visual cues, such as picture showing high-calorie food or alcoholic beverages, can be directly manipulated through the regulation, i.e., “pushed away” through successful down-regulation or “dragged towards oneself” through up-regulation (or non-regulation). Providing such approach and avoidance consequences of neural self-regulation may increase the likelihood that learned strategies are transferred to a natural environment. The importance of such motivational factors for drug addiction is demonstrated by behavioral interventions showing that making avoidance movements (pushing a joystick) in response to alcohol pictures can change the automatic approach bias and improve treatment outcomes of alcohol-dependent patients (Wiers et al., 2011; Eberl et al., 2013).

On the other hand, the present paradigm allows participants to directly manipulate and control the visual cue which can be predicted to increase the participant’s sense of agency and perception of self-efficacy (Bandura, 1977)—factors that are central for the maintenance of drug-abstinent behavior (Greenfield et al., 2000). One limitation of our paradigm relates to the effects that visual changes in picture size may themselves have on motivational brain responses. However, changes in picture size have been shown to affect neural responses of emotional brain networks only marginally (De Cesarei and Codispoti, 2006). More importantly, in the present paradigm such effects are also controlled for by the mirror runs, which repeat the size changes of the regulation run. Nonetheless, care should be taken when defining maximal and minimum picture sizes for a specific scanner-projector set-up in order to remain within a visible range.

### Summary and conclusions

The visual feedback subsystem we have developed has been tailored to specifically allow (i) feedback-guided regulation of visual cue reactivity, i.e., to control brain responses to pictorial cues *during* exposure to those cues; and (ii) to increase motivational relevance of visual feedback by using decreases (avoidance) or increases (approach) of stimulus size as an indication of successful or unsuccessful down-regulation. Further, the feedback subsystem is able to adapt to peoples’ individual neurophysiological response range. These individual ranges are scaled to fall within a standard range before being mapped to the corresponding image sizes for display. Importantly, the introduction of the mirror run controls for physical/perceptual confounds, allowing separation of BOLD response changes resulting from successful regulation from those related to variations of visual stimulus properties or visual responses, such as habituation. Finally, the present feedback subsystem displays a new food image size every 2 s allowing good performance and providing a minimum delay in line with existing visual feedback subsystems (Weiskopf et al., 2003, 2004; Sitaram et al., 2007b, 2008). To conclude, our approach may facilitate the control of brain activation during neurofeedback training involving simultaneous presentation of visual cues and may thus help the translation of neurofeedback into clinical applications, such as the regulation of craving responses to substance-related visual cues in addictive disorders.

## Conflict of interest statement

The authors declare that the research was conducted in the absence of any commercial or financial relationships that could be construed as a potential conflict of interest.
